# President’s Address

**Published:** 1901-02

**Authors:** T. V. Hubbard

**Affiliations:** Atlanta, Ga.


ATLANTA

### Journal-Record of Medicine.

Successor to Atlanta Medical and Surgical Journal, Established 1855, and Southern Medical Record, Established 1870.

VOL. II. FEBRUARY, 1901. No. 11.

BERNARD WOLFF, M.D., EDITOR. M. B. HUTCHINS, M.D., BUSINESS MANAGER.

Published Monthly. Office No. 64 Marietta Street, Opposite Post-office.

### ORIGINAL COMMUNICATIONS.

### PRESIDENT'S ADDRESS.

BY T. V. HUBBARD, M.D.,
ATLANTA, GA.

*Gentlemen of the Atlanta Society of Medicine:*

In assuming the duties and responsibilities that will devolve upon me as president of this society for the ensuing year, I am but following a time honored custom in suggesting what seems to me to be the best method of increasing our membership, intensifying individual interest, and broadening the scope and usefulness of this organization to the medical profession of Atlanta.

What I have to say is offered merely as suggestions, subject to the approval or disapproval of the society, and I will at all times be glad to receive suggestions from any member who has a feasible plan whereby the interest in this organization may be increased.

In succeeding our honored and retiring president, I recognize the fact that it is no small undertaking to keep up the progress he has so intelligently initiated, and in attempting to do so, I shall expect the hearty cooperation and undivided support of every member of this society, for "in union there is strength," and without a broad minded, well directed effort, any society of this character must inevitably fail. I sincerely hope that the members will not assume the faulty position that the work must all be done by the officers, for, from a scientific standpoint, they may constitute the least important part of its membership.

The work so enthusiastically inaugurated, and so energetically pursued by our retiring president and his committee in prosecuting the Osteopathists, quacks and other unlicensed and illegitimate practitioners, should receive the highest praise, and an earnest request made for a continuance of their laudable work.

I would suggest in this connection, the employment of an attorney at a small remuneration, to push the cases now in court to a successful issue, and to look after any other legal questions with which the society might become involved.

For a city the size of Atlanta, and for the number of physicians here, it must be admitted our membership is entirely too small; and to my mind the most effective way of increasing our number is for each member to constitute himself a committee of one, and see that he induces at least one physician to join us in 1901.

I think an amendment to the by-laws creating an honorary list of membership would be a step in the right direction, and this list should include those who from age or ill health have retired from practice, and those who are absent from the city, but pursuing their honorable profession in some other part of the world.

The chief feature which renders the meeting of a body of medical men most successful, is a varied and well arranged program. The work of getting up papers should not devolve entirely upon the president and secretary, and it is my purpose to appoint a committee of three, whose business it shall be to arrange a program for each meeting. They should secure the promise from some member for a paper at each meeting, and appoint another member to open the discussion, notifying him in advance of the subject matter to be discussed.

Another feature which it is my purpose to especially encourage, and I hope will meet with prompt response from the members, is a more extensive report of cases. From the number of members we have at present, there should never a meeting pass without a report of some interesting and unique case. In other words, I think the society should be made more clinical in its character, and encourage the members to show their results of medical treatment and surgical operations, and demonstrate any mechanical appliance which may be useful to the profession.

The demand upon a physician's time is such that it is frequently impossible to prepare a scientific paper, but he can report an interesting case and elicit a broad discussion and exchange of views, which, after all, gives the best result from an educational standpoint. This is especially beneficial to the younger members, who can in this way acquire the ability to debate scientific questions accurately. I indulge the hope that the reporting of successful cases will not entirely supplant an occasional acknowledgement of our mistakes, which are so necessary to the progress of medical science.

The fact that some physicians have a better command of English and a broader familiarity with medical terms than others, should in no wise keep the latter from giving us the benefit of their experience with rare cases.

I have been informed by the Library Committee that the society will have a hall in the new Carnegie Library, in which to hold our regular meetings. This, I think, is cause for sincere congratulation, especially in view of the fact that there was so much opposition to the expenditure of our funds for this purpose.

The securing of a place of meeting is *the thing* most to be desired, if we expect the continuation of our society, and it is to be regretted that we have not ere this a permanent home. I think to a great extent it accounts for the lack of interest manifested for the past few years. It must be confessed with shame that there is a certain amount of discord in the medical profession of our city, and to dispel this discord is a consummation devoutly to be hoped for. To accomplish this result the physicians should meet oftener socially, become better acquainted and exchange their views on the interesting topics of the day, as well as matters connected with their profession, and I sincerely hope we may see this when we get a pleasant and permanent place of meeting.

The society in the past has not accomplished that amount of work in the way of original research and investigation which should characterize an organization of its size and importance. Let us hope during this year that some members at least will come forward and give us the result of their investigation of some original question.

That the laity, as well as a large number of physicians, are unfamiliar with our code of ethics and the rules which govern our conduct towards them and one another, is too well known to admit of discussion. It has often occurred to me that the promulgation of such knowledge through the medium of the daily press, at stated intervals during the year, would relieve both physician and patient of a great deal of embarrassment and redound to the dignity of the one and the benefit of the other, providing, of course, such publication could be secured gratuitously. In making this suggestion, I refer simply to those sections of the code describing the conduct of physicians towards each other, the relation of physicians to patients and patients to physicians, and the relation of physicians to the public.

In conclusion, gentlemen, allow me to thank you for the honor you have conferred upon me by making me your presiding officer for one year, and I will show my appreciation in a more substantial way by an earnest effort to do my duty. At the end of my term, if my administration can compare favorably with that of the distinguished gentlemen who have preceded me, it will be no mean consolation.

